# *rac*-4a,10b-*cis*,10b,5c-*trans*-5-(7-Methyl-2-oxo-2*H*-chromen-4-yl)-3,4,4a,5,6,10b-hexa­hydro-2*H*-pyrano[3,2-*c*]quinoline

**DOI:** 10.1107/S1600536813001876

**Published:** 2013-01-23

**Authors:** M. Kayalvizhi, G. Vasuki, Shriniwas D. Samant, Kailas K. Sanap

**Affiliations:** a Department of Physics, Kunthavai Naachiar Government Arts College (W) (Autonomous), Thanjavur-7, India; b Department of Chemistry, Institute of Chemical Technology, N.M. Parekh Road, Matunga, Mumbai 400 019, India

## Abstract

In the racemic title compound, C_22_H_21_NO_3_, the nitro­gen-containing ring of the pyran­oquinoline moiety adopts a slightly distorted half-chair conformation and the oxygen-containing ring adopts a slightly distorted chair conformation. The benzene rings make a dihedral angle of 84.97 (8)°. In the crystal, weak C—H⋯O inter­actions link the mol­ecules into chains extending along the *a*-axis direction.

## Related literature

For general background and related coumarin compounds, see: Aazam *et al.* (2006 ▶); Chinnakali *et al.* (2009 ▶); Du *et al.* (2010 ▶); Pereira Silva *et al.* (2010 ▶). For ring conformational analysis, see: Cremer & Pople (1975 ▶).
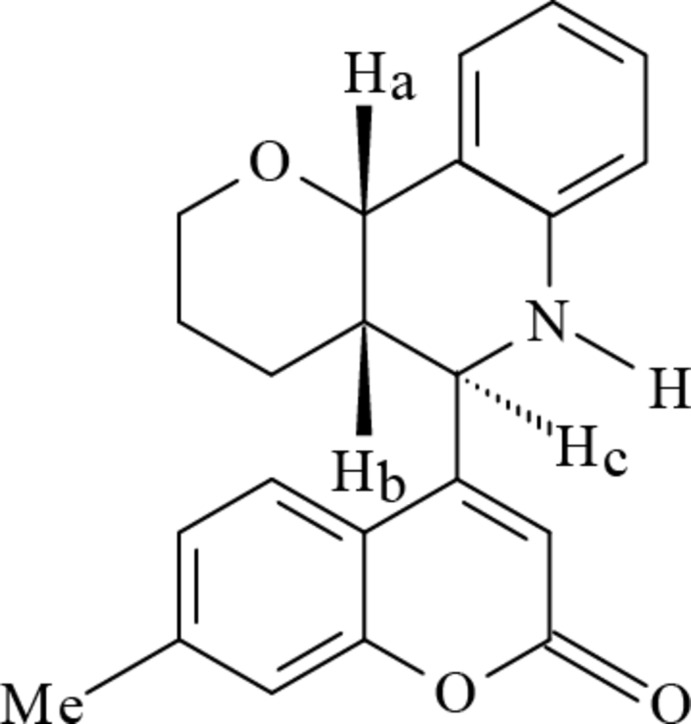


## Experimental

### 

#### Crystal data

C_22_H_21_NO_3_
*M* *_r_* = 347.40
Triclinic, 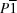

*a* = 7.7529 (4) Å
*b* = 11.2790 (7) Å
*c* = 11.7563 (11) Å
α = 117.232 (3)°
β = 98.475 (3)°
γ = 101.301 (2)°
*V* = 862.60 (11) Å^3^
*Z* = 2
Mo *K*α radiation
μ = 0.09 mm^−1^
*T* = 296 K
0.20 × 0.15 × 0.15 mm

#### Data collection

Bruker Kappa APEXII CCD diffractometer
Absorption correction: multi-scan (*SADABS*; Bruker 1999 ▶) *T*_min_ = 0.984, *T*_max_ = 0.987
18945 measured reflections
5009 independent reflections
3544 reflections with *I* > 2σ(*I*)
*R*_int_ = 0.030

#### Refinement

*R*[*F* ^2^ > 2σ(*F* ^2^)] = 0.055
*wR*(*F* ^2^) = 0.177
*S* = 1.03
5009 reflections
235 parameters
H-atom parameters constrained
Δρ_max_ = 0.41 e Å^−3^
Δρ_min_ = −0.27 e Å^−3^

### 

Data collection: *APEX2* (Bruker, 2004 ▶); cell refinement: *APEX2* and *SAINT* (Bruker, 2004 ▶); data reduction: *SAINT* and *XPREP* (Bruker, 2004 ▶); program(s) used to solve structure: *SHELXS97* (Sheldrick, 2008 ▶); program(s) used to refine structure: *SHELXL97* (Sheldrick, 2008 ▶); molecular graphics: *ORTEP-3* (Farrugia, 2012 ▶); software used to prepare material for publication: *PLATON* (Spek, 2009 ▶).

## Supplementary Material

Crystal structure: contains datablock(s) I, global. DOI: 10.1107/S1600536813001876/zs2247sup1.cif

Structure factors: contains datablock(s) I. DOI: 10.1107/S1600536813001876/zs2247Isup2.hkl

Additional supplementary materials:  crystallographic information; 3D view; checkCIF report

## Figures and Tables

**Table 1:** Hydrogen-bond geometry (Å, °)

*D*—H⋯*A*	*D*—H	H⋯*A*	*D*⋯*A*	*D*—H⋯*A*
C12—H12*A*⋯O1^i^	0.97	2.59	3.307 (3)	131
C20—H20⋯O2^ii^	0.93	2.40	3.275 (2)	157

Symmetry codes: (i) 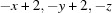; (ii) 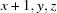.

**Fig. 1.**
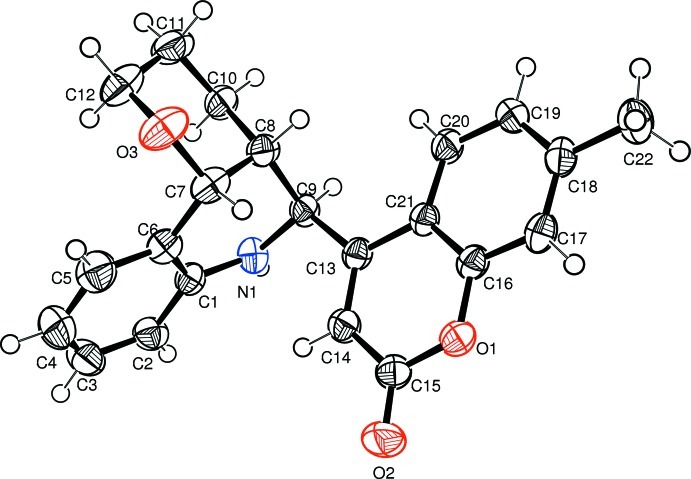
The molecular structure of the title compound showing atom numbering, with displacement ellipsoids drawn at the 50% probability level.

**Fig. 2.**
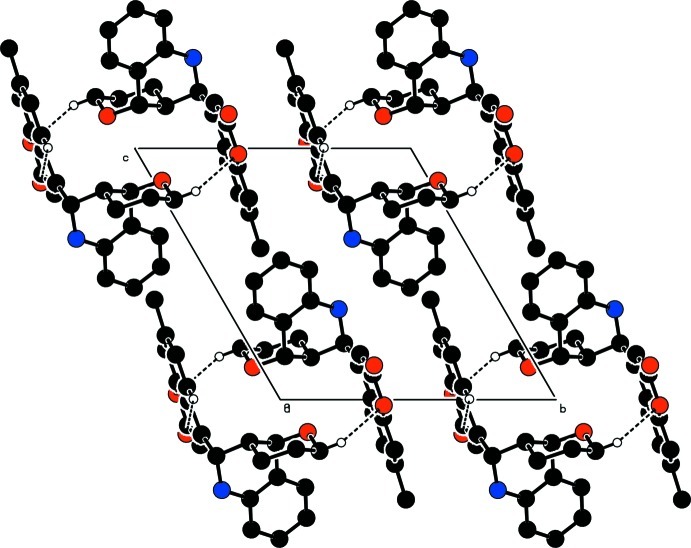
A view of the crystal packing of the title compound looking down the a axis, showing C—H···O interactions as dashed lines.

## supplementary crystallographic information

### Comment

Coumarin is the simplest member of the group of oxygen heterocyclic compounds called benzo-2-pyrones. Coumarins are an important class of compound due to their presence in natural products as well as their medicinal applications, e.g. as anti-inflammatory, anti-viral, antioxidant, antibacterial, antifungal, anti-HIV and as anti-carcinogenic agents (Pereira Silva *et al.*, 2010). Coumarin and its derivatives also have applications as fluorescent dyes for synthetic fibres and daylight fluorescent pigments (Aazam *et al.*, 2006) and as cosmetics, optical brightening agents and laser dyes (Pereira Silva *et al.*, 2010). The synthesis of pyranoquinoline derivatives has been the focus of great interest, because it was reported that these possess a broad spectrum of biological properties such as psychotropic activity and anti-allergenic activity and they are also used for the treatment of proliferative diseases, such as cancer (Du *et al.*, 2010). Compounds containing pyranoquinolone motifs also exhibit antiproliferative and antitubulin activities and it includes antibacterial and antifungal activities. Some of the pyranoquinoline derivatives have been found to block acetylcholinesterase and cell calcium signals, and cause neuroprotection against calcium overload and free radicals (Chinnakali *et al.*, 2009).

We report herein the crystal structure of the racemic title compound, a pyranoquinoline-substituted methyl coumarin derivative, C_22_H_21_NO_3_ (Fig. 1). The dihedral angle between the phenyl rings of the coumarin molecule and the pyranoquinoline moiety is 84.97 (8)° . The C15 atom of the carbonyl group has a distorted trigonal geometry with O2—C15—O1 [117.36 (14)°] and O2—C15—C14 [125.26 (16)°], deviating significantly from the ideal *sp*^2^ value of 120°, which is consistent with the values observed in a related structure (Pereira Silva *et al.*, 2010). In the crystal, weak intermolecular C20—H···O2^ii^ hydrogen bonds together with C12—H···O1^i^ hydrogen bonds between inversion-related molecules (Table 1), give one-dimensional chain structures which extend along the *a* axis (Fig. 2). Present also in the crystal packing are C5—H···π ring interactions [minimum C···*Cg* separation, 3.910 (3) Å] (for symmetry codes, see Table 1). The substituent ring defined by (N1, C1, C6–C9) adopts a slightly distorted half-chair conformation with Q = 0.4852 (18) Å, θ = 48.0 (2)° and φ = 259.3 (3)° while the ring defined by (O3, C7–C12) adopts a slightly distorted chair conformation with Q = 0.548 (2) Å, θ = 2.8 (2)° and φ = 300 (5)° (Cremer & Pople, 1975).

### Experimental

7-Methylcoumarin-4-azadiene (0.263 g, 1 mmol) and ZnCl_2_ (0.136 g, 1 mmol) were stirred in dichloroethane (5 ml) for 15 minutes and dihydropyran (0.252 g, 3 mmol) was added slowly at room temperature. The solution was heated till complete consumption of the coumarin reagent. The solution was cooled to room temperature, quenched with water and the product was extracted with chloroform. The extract was dried over anhydrous Na_2_SO_4_ and the solvent evaporated to obtain a sticky mass which was purified by column chromatography on silica gel using chloroform.

### Refinement

All the H atoms were positioned geometrically and treated as riding on their parent atoms, with N—H = 0.86 Å, C—H = 0.93 Å (aromatic), 0.96 Å (methyl) and 0.97 Å (methylene), and refined using a riding model with *U*_iso_(H) = 1.2*U*_eq_ or 1.5 *U*_eq_ (parent atom).

### Crystal data

**Table:**

C_22_H_21_NO_3_	*Z* = 2
*M**_r_* = 347.40	*F*(000) = 368
Triclinic, *P*1	*D*_x_ = 1.338 Mg m^−^^3^
Hall symbol: -P 1	Mo *K*α radiation, λ = 0.71073 Å
*a* = 7.7529 (4) Å	Cell parameters from 6950 reflections
*b* = 11.2790 (7) Å	θ = 2.1–30.2°
*c* = 11.7563 (11) Å	µ = 0.09 mm^−^^1^
α = 117.232 (3)°	*T* = 296 K
β = 98.475 (3)°	Block, colourless
γ = 101.301 (2)°	0.20 × 0.15 × 0.15 mm
*V* = 862.60 (11) Å^3^	

### Data collection

**Table:**

Bruker Kappa APEXII CCD diffractometer	5009 independent reflections
Radiation source: fine-focus sealed tube	3544 reflections with *I* > 2σ(*I*)
Graphite monochromator	*R*_int_ = 0.030
ω and φ scan	θ_max_ = 30.2°, θ_min_ = 2.0°
Absorption correction: multi-scan (*SADABS*; Bruker 1999)	*h* = −10→10
*T*_min_ = 0.984, *T*_max_ = 0.987	*k* = −15→15
18945 measured reflections	*l* = −16→16

### Refinement

**Table:**

Refinement on *F*^2^	Primary atom site location: structure-invariant direct methods
Least-squares matrix: full	Secondary atom site location: difference Fourier map
*R*[*F*^2^ > 2σ(*F*^2^)] = 0.055	Hydrogen site location: inferred from neighbouring sites
*wR*(*F*^2^) = 0.177	H-atom parameters constrained
*S* = 1.03	*w* = 1/[σ^2^(*F*_o_^2^) + (0.0832*P*)^2^ + 0.3474*P*] where *P* = (*F*_o_^2^ + 2*F*_c_^2^)/3
5009 reflections	(Δ/σ)_max_ = 0.001
235 parameters	Δρ_max_ = 0.41 e Å^−^^3^
0 restraints	Δρ_min_ = −0.27 e Å^−^^3^

### Special details

**Table:**

Geometry. All e.s.d.'s (except the e.s.d. in the dihedral angle between two l.s. planes) are estimated using the full covariance matrix. The cell e.s.d.'s are taken into account individually in the estimation of e.s.d.'s in distances, angles and torsion angles; correlations between e.s.d.'s in cell parameters are only used when they are defined by crystal symmetry. An approximate (isotropic) treatment of cell e.s.d.'s is used for estimating e.s.d.'s involving l.s. planes.
Refinement. Refinement of *F*^2^ against ALL reflections. The weighted *R*-factor *wR* and goodness of fit *S* are based on *F*^2^, conventional *R*-factors *R* are based on *F*, with *F* set to zero for negative *F*^2^. The threshold expression of *F*^2^ > σ(*F*^2^) is used only for calculating *R*-factors(gt) *etc*. and is not relevant to the choice of reflections for refinement. *R*-factors based on *F*^2^ are statistically about twice as large as those based on *F*, and *R*- factors based on ALL data will be even larger.

### Fractional atomic coordinates and isotropic or equivalent isotropic displacement parameters (Å^2^)

**Table:**

	*x*	*y*	*z*	*U*_iso_*/*U*_eq_	
O1	0.69639 (15)	0.63713 (13)	0.02296 (11)	0.0385 (3)	
C13	0.9273 (2)	0.64223 (15)	−0.13810 (14)	0.0305 (3)	
C21	1.0013 (2)	0.66621 (15)	−0.00600 (14)	0.0306 (3)	
N1	0.95359 (19)	0.59869 (13)	−0.35976 (12)	0.0343 (3)	
H1	0.9425	0.5149	−0.4199	0.041*	
C16	0.8806 (2)	0.66458 (16)	0.07070 (15)	0.0321 (3)	
C14	0.7463 (2)	0.61512 (17)	−0.18120 (16)	0.0363 (3)	
H14	0.6982	0.5978	−0.2665	0.044*	
C9	1.0532 (2)	0.65038 (16)	−0.22402 (14)	0.0320 (3)	
H9	1.1325	0.5922	−0.2253	0.038*	
C1	0.8757 (2)	0.68235 (16)	−0.39430 (14)	0.0315 (3)	
C17	0.9383 (2)	0.69114 (17)	0.19930 (16)	0.0377 (3)	
H17	0.8538	0.6898	0.2476	0.045*	
C18	1.1206 (2)	0.71958 (17)	0.25613 (16)	0.0375 (3)	
C19	1.2429 (2)	0.71608 (18)	0.17925 (17)	0.0394 (4)	
H19	1.3659	0.7322	0.2154	0.047*	
C20	1.1850 (2)	0.68931 (18)	0.05130 (16)	0.0372 (3)	
H20	1.2691	0.6865	0.0020	0.045*	
O3	1.16897 (19)	1.03131 (13)	−0.12889 (13)	0.0526 (4)	
C8	1.1758 (2)	0.80019 (17)	−0.16807 (15)	0.0363 (3)	
H8	1.2389	0.8366	−0.0748	0.044*	
O2	0.45952 (18)	0.58805 (17)	−0.13623 (15)	0.0564 (4)	
C15	0.6228 (2)	0.61153 (18)	−0.10156 (17)	0.0377 (3)	
C7	1.0589 (2)	0.89261 (16)	−0.17287 (16)	0.0384 (4)	
H7	0.9858	0.9001	−0.1100	0.046*	
C6	0.9262 (2)	0.82680 (16)	−0.30839 (16)	0.0359 (3)	
C10	1.3203 (2)	0.8037 (2)	−0.24303 (18)	0.0419 (4)	
H10A	1.4040	0.7555	−0.2280	0.050*	
H10B	1.2614	0.7557	−0.3376	0.050*	
C2	0.7472 (2)	0.62255 (19)	−0.51719 (17)	0.0415 (4)	
H2	0.7121	0.5264	−0.5744	0.050*	
C22	1.1856 (3)	0.7542 (2)	0.39765 (18)	0.0518 (5)	
H22A	1.3155	0.7709	0.4209	0.078*	
H22B	1.1263	0.6775	0.4072	0.078*	
H22C	1.1566	0.8365	0.4553	0.078*	
C12	1.2970 (3)	1.03446 (19)	−0.2043 (2)	0.0537 (5)	
H12A	1.3662	1.1307	−0.1712	0.064*	
H12B	1.2311	0.9952	−0.2965	0.064*	
C5	0.8472 (3)	0.9065 (2)	−0.3492 (2)	0.0514 (5)	
H5	0.8796	1.0026	−0.2928	0.062*	
C4	0.7226 (3)	0.8466 (3)	−0.4707 (3)	0.0616 (6)	
H4	0.6724	0.9019	−0.4964	0.074*	
C3	0.6725 (3)	0.7046 (2)	−0.5542 (2)	0.0542 (5)	
H3	0.5874	0.6638	−0.6362	0.065*	
C11	1.4262 (3)	0.9538 (2)	−0.1964 (2)	0.0541 (5)	
H11A	1.5002	0.9976	−0.1055	0.065*	
H11B	1.5073	0.9547	−0.2517	0.065*	

### Atomic displacement parameters (Å^2^)

**Table:**

	*U*^11^	*U*^22^	*U*^33^	*U*^12^	*U*^13^	*U*^23^
O1	0.0341 (6)	0.0493 (7)	0.0405 (6)	0.0154 (5)	0.0186 (5)	0.0257 (5)
C13	0.0329 (7)	0.0325 (7)	0.0300 (7)	0.0116 (6)	0.0133 (6)	0.0168 (6)
C21	0.0330 (7)	0.0326 (7)	0.0307 (7)	0.0112 (6)	0.0127 (6)	0.0179 (6)
N1	0.0461 (8)	0.0300 (6)	0.0254 (6)	0.0145 (5)	0.0118 (5)	0.0110 (5)
C16	0.0333 (8)	0.0330 (7)	0.0347 (7)	0.0117 (6)	0.0140 (6)	0.0188 (6)
C14	0.0354 (8)	0.0440 (9)	0.0344 (8)	0.0131 (6)	0.0123 (6)	0.0221 (7)
C9	0.0336 (8)	0.0388 (8)	0.0285 (7)	0.0134 (6)	0.0134 (6)	0.0185 (6)
C1	0.0342 (8)	0.0345 (7)	0.0290 (7)	0.0109 (6)	0.0135 (6)	0.0166 (6)
C17	0.0450 (9)	0.0409 (8)	0.0358 (8)	0.0145 (7)	0.0202 (7)	0.0227 (7)
C18	0.0469 (9)	0.0359 (8)	0.0328 (7)	0.0104 (7)	0.0119 (6)	0.0202 (6)
C19	0.0349 (8)	0.0485 (9)	0.0399 (8)	0.0119 (7)	0.0084 (6)	0.0270 (8)
C20	0.0350 (8)	0.0480 (9)	0.0387 (8)	0.0145 (7)	0.0162 (6)	0.0270 (7)
O3	0.0595 (8)	0.0312 (6)	0.0472 (7)	0.0018 (5)	0.0232 (6)	0.0061 (5)
C8	0.0350 (8)	0.0436 (9)	0.0263 (7)	0.0046 (6)	0.0087 (6)	0.0170 (6)
O2	0.0334 (7)	0.0876 (11)	0.0619 (9)	0.0234 (7)	0.0187 (6)	0.0447 (8)
C15	0.0333 (8)	0.0444 (9)	0.0429 (8)	0.0150 (6)	0.0142 (6)	0.0253 (7)
C7	0.0426 (9)	0.0307 (7)	0.0318 (7)	0.0051 (6)	0.0161 (6)	0.0082 (6)
C6	0.0373 (8)	0.0332 (8)	0.0372 (8)	0.0117 (6)	0.0148 (6)	0.0158 (6)
C10	0.0372 (9)	0.0538 (10)	0.0450 (9)	0.0143 (7)	0.0179 (7)	0.0307 (8)
C2	0.0387 (9)	0.0463 (9)	0.0346 (8)	0.0095 (7)	0.0088 (7)	0.0179 (7)
C22	0.0595 (12)	0.0614 (12)	0.0362 (9)	0.0124 (9)	0.0112 (8)	0.0287 (9)
C12	0.0603 (12)	0.0368 (9)	0.0536 (11)	0.0000 (8)	0.0229 (9)	0.0179 (8)
C5	0.0519 (11)	0.0411 (9)	0.0682 (13)	0.0204 (8)	0.0198 (9)	0.0294 (9)
C4	0.0565 (13)	0.0700 (14)	0.0808 (15)	0.0291 (11)	0.0165 (11)	0.0524 (13)
C3	0.0425 (10)	0.0737 (14)	0.0523 (11)	0.0169 (9)	0.0072 (8)	0.0381 (11)
C11	0.0416 (10)	0.0625 (12)	0.0491 (10)	−0.0022 (8)	0.0143 (8)	0.0271 (9)

### Geometric parameters (Å, º)

**Table:**

O1—C15	1.362 (2)	C8—C7	1.523 (2)
O1—C16	1.3720 (19)	C8—C10	1.529 (2)
C13—C14	1.342 (2)	C8—H8	0.9800
C13—C21	1.451 (2)	O2—C15	1.207 (2)
C13—C9	1.5237 (19)	C7—C6	1.511 (2)
C21—C16	1.3948 (19)	C7—H7	0.9800
C21—C20	1.401 (2)	C6—C5	1.391 (2)
N1—C1	1.386 (2)	C10—C11	1.522 (3)
N1—C9	1.4463 (19)	C10—H10A	0.9700
N1—H1	0.8600	C10—H10B	0.9700
C16—C17	1.382 (2)	C2—C3	1.373 (3)
C14—C15	1.442 (2)	C2—H2	0.9300
C14—H14	0.9300	C22—H22A	0.9600
C9—C8	1.536 (2)	C22—H22B	0.9600
C9—H9	0.9800	C22—H22C	0.9600
C1—C2	1.398 (2)	C12—C11	1.499 (3)
C1—C6	1.398 (2)	C12—H12A	0.9700
C17—C18	1.377 (2)	C12—H12B	0.9700
C17—H17	0.9300	C5—C4	1.373 (3)
C18—C19	1.397 (2)	C5—H5	0.9300
C18—C22	1.502 (2)	C4—C3	1.372 (3)
C19—C20	1.374 (2)	C4—H4	0.9300
C19—H19	0.9300	C3—H3	0.9300
C20—H20	0.9300	C11—H11A	0.9700
O3—C12	1.431 (2)	C11—H11B	0.9700
O3—C7	1.4316 (19)		
			
C15—O1—C16	121.40 (12)	O1—C15—C14	117.37 (14)
C14—C13—C21	118.25 (13)	O3—C7—C6	112.52 (14)
C14—C13—C9	121.18 (13)	O3—C7—C8	111.57 (14)
C21—C13—C9	120.56 (13)	C6—C7—C8	111.58 (12)
C16—C21—C20	116.62 (13)	O3—C7—H7	106.9
C16—C21—C13	117.98 (13)	C6—C7—H7	106.9
C20—C21—C13	125.39 (13)	C8—C7—H7	106.9
C1—N1—C9	120.93 (12)	C5—C6—C1	118.48 (16)
C1—N1—H1	119.5	C5—C6—C7	121.39 (15)
C9—N1—H1	119.5	C1—C6—C7	120.10 (14)
O1—C16—C17	115.72 (13)	C11—C10—C8	110.52 (15)
O1—C16—C21	121.83 (13)	C11—C10—H10A	109.5
C17—C16—C21	122.45 (15)	C8—C10—H10A	109.5
C13—C14—C15	123.12 (14)	C11—C10—H10B	109.5
C13—C14—H14	118.4	C8—C10—H10B	109.5
C15—C14—H14	118.4	H10A—C10—H10B	108.1
N1—C9—C13	112.47 (12)	C3—C2—C1	120.53 (17)
N1—C9—C8	108.27 (12)	C3—C2—H2	119.7
C13—C9—C8	112.04 (12)	C1—C2—H2	119.7
N1—C9—H9	108.0	C18—C22—H22A	109.5
C13—C9—H9	108.0	C18—C22—H22B	109.5
C8—C9—H9	108.0	H22A—C22—H22B	109.5
N1—C1—C2	119.91 (14)	C18—C22—H22C	109.5
N1—C1—C6	120.76 (14)	H22A—C22—H22C	109.5
C2—C1—C6	119.33 (15)	H22B—C22—H22C	109.5
C18—C17—C16	120.12 (14)	O3—C12—C11	111.81 (16)
C18—C17—H17	119.9	O3—C12—H12A	109.3
C16—C17—H17	119.9	C11—C12—H12A	109.3
C17—C18—C19	118.40 (14)	O3—C12—H12B	109.3
C17—C18—C22	120.50 (15)	C11—C12—H12B	109.3
C19—C18—C22	121.10 (16)	H12A—C12—H12B	107.9
C20—C19—C18	121.31 (15)	C4—C5—C6	121.58 (18)
C20—C19—H19	119.3	C4—C5—H5	119.2
C18—C19—H19	119.3	C6—C5—H5	119.2
C19—C20—C21	120.98 (14)	C3—C4—C5	119.68 (18)
C19—C20—H20	119.5	C3—C4—H4	120.2
C21—C20—H20	119.5	C5—C4—H4	120.2
C12—O3—C7	112.87 (13)	C4—C3—C2	120.39 (18)
C7—C8—C10	110.95 (13)	C4—C3—H3	119.8
C7—C8—C9	109.85 (13)	C2—C3—H3	119.8
C10—C8—C9	111.42 (13)	C12—C11—C10	110.22 (15)
C7—C8—H8	108.2	C12—C11—H11A	109.6
C10—C8—H8	108.2	C10—C11—H11A	109.6
C9—C8—H8	108.2	C12—C11—H11B	109.6
O2—C15—O1	117.36 (14)	C10—C11—H11B	109.6
O2—C15—C14	125.26 (16)	H11A—C11—H11B	108.1
			
C14—C13—C21—C16	−2.2 (2)	C13—C9—C8—C10	171.57 (13)
C9—C13—C21—C16	176.72 (13)	C16—O1—C15—O2	179.89 (15)
C14—C13—C21—C20	176.89 (15)	C16—O1—C15—C14	−0.3 (2)
C9—C13—C21—C20	−4.2 (2)	C13—C14—C15—O2	179.75 (18)
C15—O1—C16—C17	178.94 (14)	C13—C14—C15—O1	0.0 (2)
C15—O1—C16—C21	−0.7 (2)	C12—O3—C7—C6	−68.9 (2)
C20—C21—C16—O1	−177.22 (14)	C12—O3—C7—C8	57.39 (19)
C13—C21—C16—O1	2.0 (2)	C10—C8—C7—O3	−52.89 (17)
C20—C21—C16—C17	3.2 (2)	C9—C8—C7—O3	−176.54 (12)
C13—C21—C16—C17	−177.65 (14)	C10—C8—C7—C6	73.92 (16)
C21—C13—C14—C15	1.3 (2)	C9—C8—C7—C6	−49.72 (16)
C9—C13—C14—C15	−177.63 (14)	N1—C1—C6—C5	178.45 (15)
C1—N1—C9—C13	81.22 (17)	C2—C1—C6—C5	−0.5 (2)
C1—N1—C9—C8	−43.13 (18)	N1—C1—C6—C7	−3.5 (2)
C14—C13—C9—N1	−11.8 (2)	C2—C1—C6—C7	177.48 (14)
C21—C13—C9—N1	169.30 (13)	O3—C7—C6—C5	−33.6 (2)
C14—C13—C9—C8	110.44 (17)	C8—C7—C6—C5	−159.93 (15)
C21—C13—C9—C8	−68.46 (18)	O3—C7—C6—C1	148.42 (14)
C9—N1—C1—C2	−165.72 (14)	C8—C7—C6—C1	22.1 (2)
C9—N1—C1—C6	15.3 (2)	C7—C8—C10—C11	51.00 (19)
O1—C16—C17—C18	179.88 (14)	C9—C8—C10—C11	173.74 (14)
C21—C16—C17—C18	−0.5 (2)	N1—C1—C2—C3	−178.30 (15)
C16—C17—C18—C19	−2.1 (2)	C6—C1—C2—C3	0.7 (2)
C16—C17—C18—C22	177.53 (16)	C7—O3—C12—C11	−59.8 (2)
C17—C18—C19—C20	2.0 (3)	C1—C6—C5—C4	−0.1 (3)
C22—C18—C19—C20	−177.66 (16)	C7—C6—C5—C4	−178.11 (18)
C18—C19—C20—C21	0.8 (3)	C6—C5—C4—C3	0.7 (3)
C16—C21—C20—C19	−3.3 (2)	C5—C4—C3—C2	−0.5 (3)
C13—C21—C20—C19	177.62 (15)	C1—C2—C3—C4	−0.2 (3)
N1—C9—C8—C7	59.54 (15)	O3—C12—C11—C10	56.8 (2)
C13—C9—C8—C7	−65.06 (16)	C8—C10—C11—C12	−52.7 (2)
N1—C9—C8—C10	−63.82 (17)		

### Hydrogen-bond geometry (Å, º)

**Table:**

*D*—H···*A*	*D*—H	H···*A*	*D*···*A*	*D*—H···*A*
C10—H10*B*···N1	0.97	2.58	2.947 (2)	103
C12—H12*A*···O1^i^	0.97	2.59	3.307 (3)	131
C14—H14···N1	0.93	2.40	2.789 (2)	105
C20—H20···O2^ii^	0.93	2.40	3.275 (2)	157
C5—H5···*Cg*5^iii^	0.93	2.98	3.910 (3)	173

Symmetry codes: (i) −*x*+2, −*y*+2, −*z*; (ii) *x*+1, *y*, *z*; (iii) −*x*+2, −*y*, −*z*.
